# Automated Manufacturing of Carbon‐11 Radiopharmaceuticals Using Cassette‐Based Radiosynthesizers

**DOI:** 10.1002/jlcr.4166

**Published:** 2025-10-06

**Authors:** Michael L. Schulte, Adam J. Rosenberg

**Affiliations:** ^1^ Medical Imaging Research Institute Indiana University School of Medicine Indianapolis Indiana USA; ^2^ Department of Radiology and Imaging Sciences Indiana University School of Medicine Indianapolis Indiana USA; ^3^ Vanderbilt University Institute of Imaging Science Nashville Tennessee USA; ^4^ Department of Radiology and Radiological Sciences Vanderbilt University Medical Center Nashville Tennessee USA; ^5^ Vanderbilt Ingram Cancer Center Nashville Tennessee USA

**Keywords:** automation, carbon‐11, cGMP, radiochemistry

## Abstract

As the field of radiochemistry moves toward the rapid development and translation of radiopharmaceuticals, the radiosynthetic methodology and manufacturing are becoming more and more refined. As the use of radiopharmaceuticals in clinical research is one of the primary goals of radiochemistry research, compliance with cGMP regulations is a key factor in radiosynthesis developments. This review is primarily focused on the automation of the radiosynthesis on modules, with a special focus on the use of disposable cassettes for the reactors and flow‐paths. This review aims to cover the developments and current state‐of‐the‐art for cassette‐based radiosynthesis of carbon‐11 radiopharmaceuticals.

## Graphical abstract

Carbon‐11 on cassette‐based radiosynthesis modules: Current state of the art.

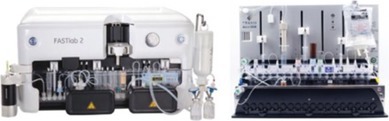

## 1 Introduction

Most PET radiotracers go through the same developmental lifecycle. When they are first discovered, they are prepared via manual methods, sometimes using semiautomated systems to reduce the radiation exposure to the operators. Once the radiotracers are advanced to research in human subjects, they are typically transferred to a fixed‐flow, fully automated system for current Good Manufacturing Process (cGMP) purposes [1]. The last stage for radiosynthesis development is translation to a cassette‐based module. Cassette‐based modules utilize a preassembled cassette, typically commercially sourced, to provide a highly reproducible process that requires minimal setup time and streamlined material sources, especially in today's rapidly changing logistics environment [2, 3].

Single‐use cassette systems can be of immense aid in this development/qualification pathway. For a fixed‐flow system, the production method needs to be optimized for each system because of the idiosyncratic differences between modules, even of the same model. For a cassette system, every facet of the production materials is identical since the cassettes are manufactured to specific tolerances. Cassette‐based synthesis offers a clear advantage over fixed‐flow systems for method sharing between production sites. Because cassettes are preassembled and standardized, they eliminate module‐to‐module variability and simplify validation, enabling straightforward replication of processes across multiple sites without extensive reoptimization. Furthermore, cassette‐based designs are straightforward to commercialize and distribute to multiple manufacturing sites. Commercial cassettes are presterilized, and all components can be manufactured under strict cGMP conditions, which greatly ease regulatory concerns. Fixed‐flow synthesizers also suffer from reusable components that not only must be thoroughly cleaned before each production (which in turn requires validation) but also suffer from wear and tear. For the purposes of this review, we have defined a “cassette‐based radiosynthesis” as an automated radiosynthesis that uses only single‐use components other than HPLC purification.

Most radiochemical methodology development has been focused on fluorine‐18 because of its commercial potential and ability to be distributed regionally (8+ h from the manufacturing site). This focus has left the methodology to install carbon‐11 underdeveloped. Carbon‐11 radiotracers are of great interest both due to their short half‐life and because isotopic substitution of carbon‐12 for carbon‐11 preserves the biological properties of the nonradioactive isotope [4]. Normally, the short half‐life (20.3 min) of carbon‐11 would be considered a detriment since it severely increases the challenge of radiosynthesis and reduces the potential length available for scanning. However, viewed from a different perspective, this is a serious benefit for scanning both animal and human research subjects as it enables scans with multiple tracers in a single day (e.g., an amyloid scan in the morning and a tau scan in the afternoon). There is growing interest in using multiple molecular imaging probes to explore related physiological changes. [5] With two or more scans in a single day, it is possible to directly compare the results of the imaging and massively simplify the logistics of the scanning, an especially important variable when it comes to human research subjects and potentially in standard of care imaging in the future.

## 2 Generation of C‐11 Synthons on Cassette

**SCHEME 1:**
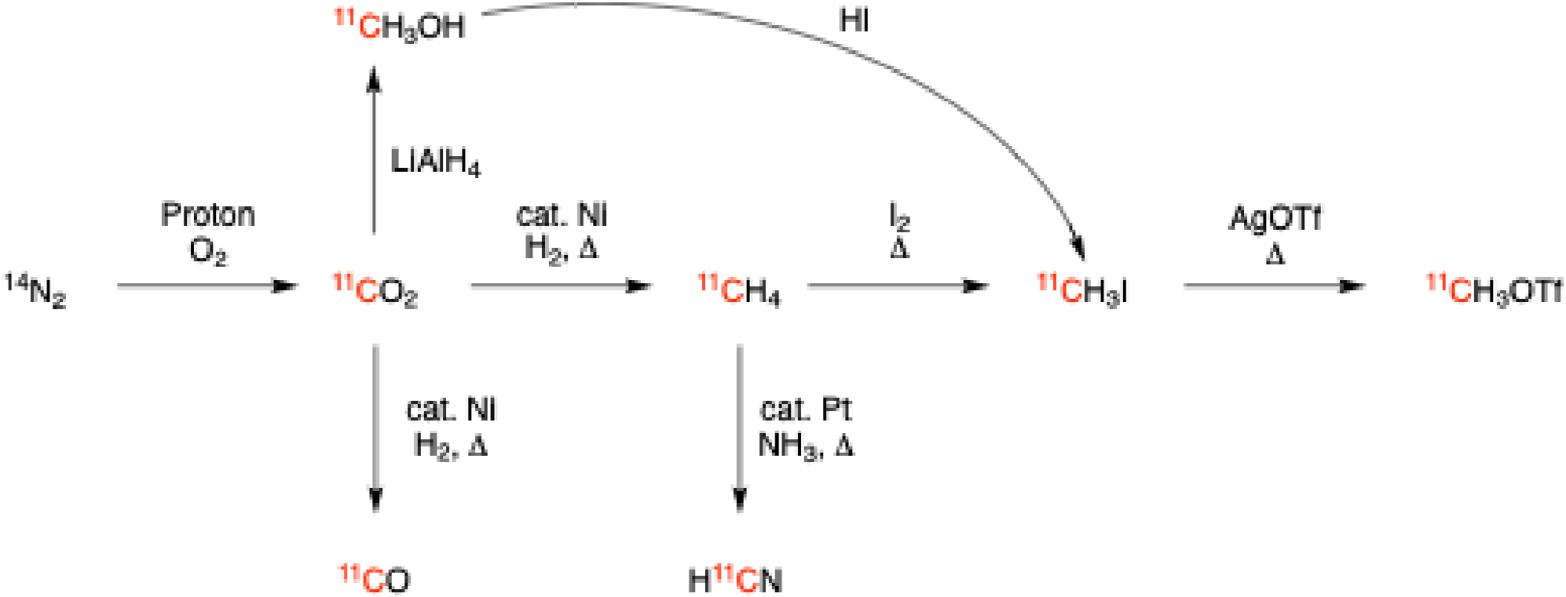
Generation of carbon‐11‐labeled synthons.

The most common method of production of carbon‐11 is via the ^14^N(*p*,α)^11^C reaction in a cyclotron gas target, followed by oxidation with oxygen to form [^11^C]CO_2_, or rarely with the use of hydrogen instead of oxygen to produce [^11^C]CH_4_. From [^11^C]CO_2_, it is possible to produce [^11^C]CO, [^11^C]CH_3_OH, [^11^C]CH_4_, [^11^C]HCN, [^11^C]MeI, and [^11^C]CH_3_OTf, with occasional forays into [^11^C]CH_2_O, [^11^C]COCl_2_, and [^11^C]CS_2_ being reported as well [6] (Scheme 1). The most common carbon‐11 synthons are [^11^C]MeI or [^11^C]MeOTf and are used either for direct nucleophilic substitution reactions or in palladium‐mediated cross‐couplings. The generation of [^11^C]MeI/MeOTf requires the use of specialized automated modules that transform the cyclotron‐produced [^11^C]CO_2_ to MeI/MeOTf using either the “wet method” of reduction to methanol and iodination with HI, or the “gas method” of reduction to methane and iodination with iodine [7, 8]. Although both methods are utilized, the gas method is used more commonly. However, the wet method is more amenable to cassette‐based methodology, avoiding the use of heated pressurized hydrogen, as well as the much higher temperatures needed for the [^11^C]CH_4_ → [^11^C]MeI conversion [7]. Although the wet method uses harsh reagents like lithium aluminum hydride and hydroiodic acid, this is ameliorated on a disposable cassette because the single‐use nature of the cassette minimizes the wear and tear concerns related to the harsh reagents and eliminates the need for cleaning the by‐products of these reagents.

To automate the wet method on a cassette, three steps need to be accounted for: (1) trapping of [^11^C]CO_2_, (2) reduction to [^11^C]CH_3_OH, and (3) functional‐group interchange to [^11^C]CH_3_I. If MeOTf is required, then a fourth step of functional‐group interchange to [^11^C]CH_3_OTf is required.

The first report of cassette automated carbon‐11 synthon generation was published in 2017 by Jolly and coworkers [9]. Using the Scintomics module series and over three cassette manifolds, they were able to generate [^11^C]CH_3_I/OTf and Label 5 different compounds. This was accomplished via the standard method of trapping on molecular sieves [10] and then heating the sieves at 275°C under a stream of nitrogen to release the purified [^11^C]CO_2_ into the second manifold. The [^11^C]CO_2_ is bubbled into a reactor containing a solution of LiAlH_4_ in THF, heating to 105°C, and removal of THF under vacuum. The [^11^C]CH_3_O^‐^ is quenched with concentrated HI, and the generated [^11^C]CH_3_I is transferred to the reaction vial through a sodium hydroxide drying column and, if required, through a silver triflate column, which has been heated to 175°C to prepare the [^11^C]CH_3_OTf. They were able to achieve up to a 35% activity yield for the final products, although the molar activity was relatively low at 18.5–33.3 GBq/μmol (500–900 Ci/mmol). Contamination with cold [^12^C]CO_2_ is a known issue with both methods, but it is a bigger issue with the wet method [11].

**FIGURE 1:**
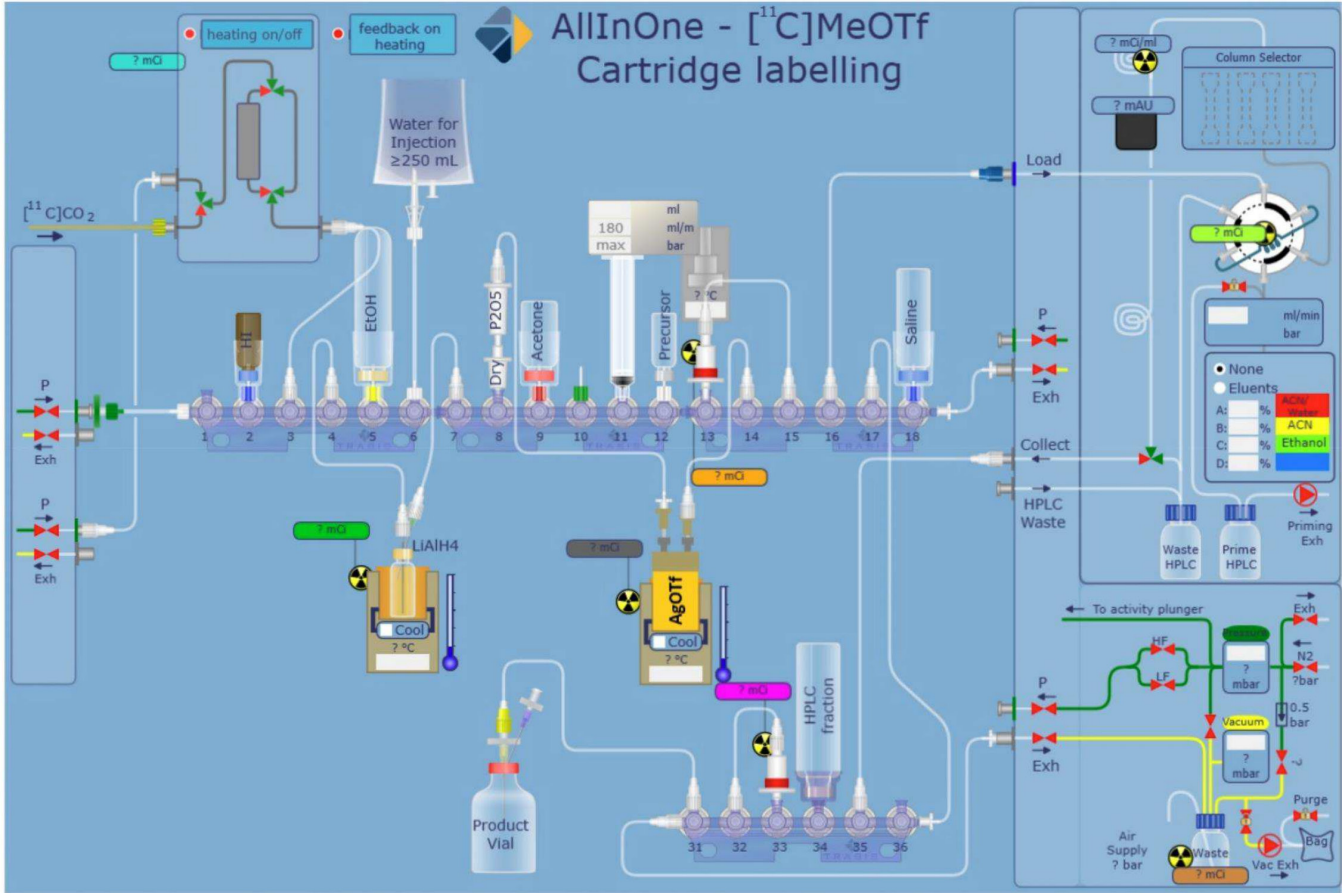
[^11^C]MeOTf generation on Trasis AIO.

More recently, in 2023, Myburgh and coworkers working with Trasis developed a fully automated cassette that used an unmodified Trasis All‐In‐One (AIO) module with a preassembled cassette to produce [^11^C]CH_3_OTf and then perform the established loop synthesis of [^11^C]PIB [12] (Figure 1). This process was reported earlier [13, 14] but not described until 2023. This process utilizes a single‐use, commercially available cassette and kit to generate the [^11^C]MeOTf via the wet method and perform the radiolabeling, HPLC purification, and reformulation to prepare [^11^C]PIB. The activity yield (from [^11^C]CO_2_) was 9.8% ± 1.7% over a 25‐min process. The molar activity achieved was only moderate at 38.9–81.1 GBq/μmol (1051–2192 Ci/mmol) but is well within the acceptance criteria for [^11^C]PIB (> 11 GBq/μmol).

## 3 Cassette‐Based Radiochemistry Modules

Radiochemistry modules based on cassette systems have revolutionized the synthesis of radiopharmaceuticals by introducing a standardized approach to radiolabeling processes [2]. Unlike fixed‐tubing or fixed‐manifold systems that require extensive setup and cleaning before and after each synthesis, cassette‐based modules utilize preassembled, disposable kits containing all required reagents and components for specific radiochemical transformations. These systems, often supported by modular hardware and automated software, have become essential tools in the production of radiotracers for molecular imaging. Their compatibility with stringent regulatory requirements, reproducibility, and ease of implementation have driven their widespread adoption in both clinical and research environments.

The core advantage of cassette‐based radiochemistry modules lies in their modularity and versatility. By enabling the rapid interchange of cassettes, these systems facilitate the synthesis of a wide array of radiotracers without the need for extensive reconfiguration of the underlying platform. This adaptability is particularly advantageous for facilities producing multiple radiopharmaceuticals, as it reduces downtime, minimizes cross‐contamination, and allows for straightforward transitions between production runs. Furthermore, the use of single‐use cassettes aligns with current good manufacturing practice (cGMP) guidelines, enhancing sterility and compliance with regulatory standards.

Another key benefit of cassette‐based systems is their high level of automation. Automated protocols reduce operator variability, improve reproducibility, and enable the synthesis of radiotracers with consistently high yields and specific activities. For isotopes like carbon‐11, with its short half‐life of ~20 min, automation is critical for meeting the stringent time constraints associated with synthesis, purification, and quality control. Additionally, the integration of software‐driven control systems allows for real‐time monitoring and documentation, streamlining compliance with regulatory agencies and facilitating batch‐to‐batch quality assurance.

Despite these advantages, cassette‐based radiochemistry modules are not without limitations. The reliance on proprietary, preassembled cassettes can lead to increased operational costs, particularly for facilities with high production demands or those developing novel radiotracers that require bespoke synthesis pathways. The design and validation of custom cassettes can be labor‐intensive and may introduce delays in radiotracer development pipelines. Moreover, the inherent constraints of cassette designs may limit the scope of chemistry that can be performed, particularly for complex or multistep syntheses requiring precise control over reaction conditions.

This review aims to provide a comprehensive analysis of cassette‐based radiochemistry modules with a particular focus on carbon‐11 radiochemistry. Although cassette‐based modules have been instrumental in expanding the accessibility of fluorine‐18 radiotracers, their utility for carbon‐11 tracer synthesis is far less explored. By examining the current state of the field, including the advantages, challenges, and future directions, this work seeks to offer insights that will guide the optimization and innovation of cassette‐based radiochemistry systems for the next generation of carbon‐11 radiopharmaceuticals.

### 3.1 Trasis AIO

**FIGURE 2:**
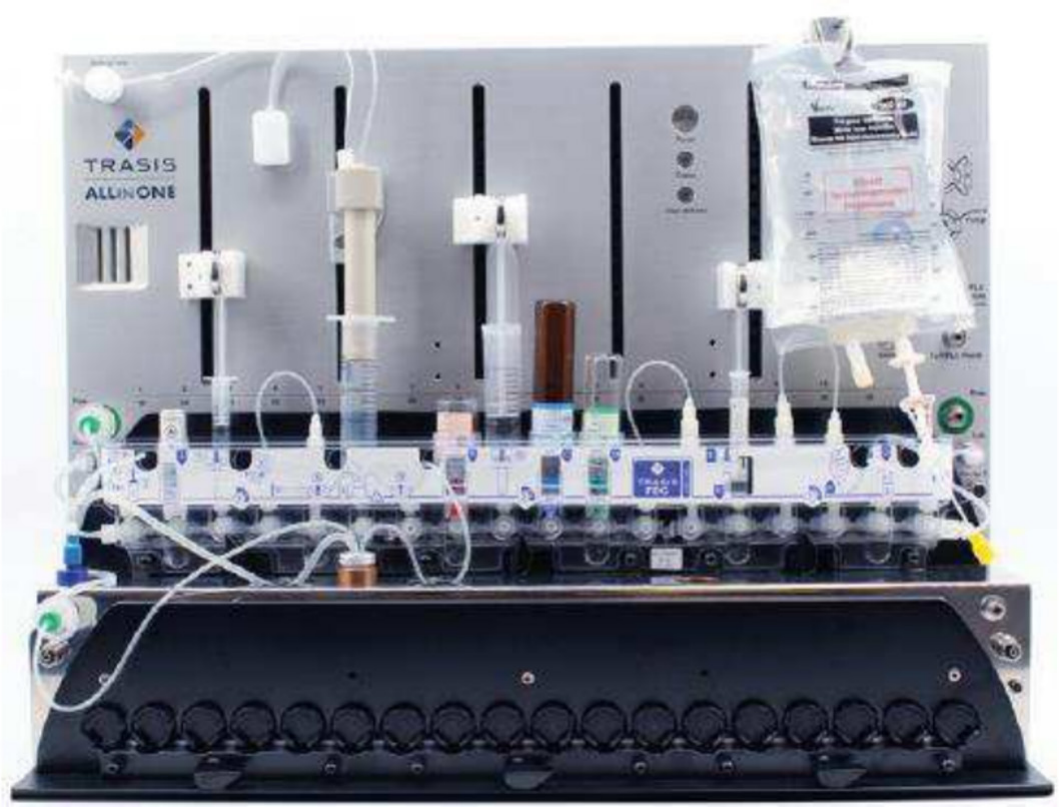
Trasis All‐In‐One module.

The AIO module from Trasis can accommodate cassettes with up to 36 valve positions and possesses multiple syringe drivers and an integrated HPLC purification system (Figure 2). It has been extensively used for fluorine‐18 chemistry [2] and somewhat for gallium‐68 labelings [15]. The AIO module has also begun to be used for cassette‐based carbon‐11 chemistry, with [^11^C]acetate [16], [^11^C]acetoacetate [17], [^11^C]methionine [13], [^11^C]choline [14], [^11^C]LY2795050 [18], and [^11^C]PIB [12] being reported on the module.

### 3.2 3D‐Printed Modules [19]

**FIGURE 3:**
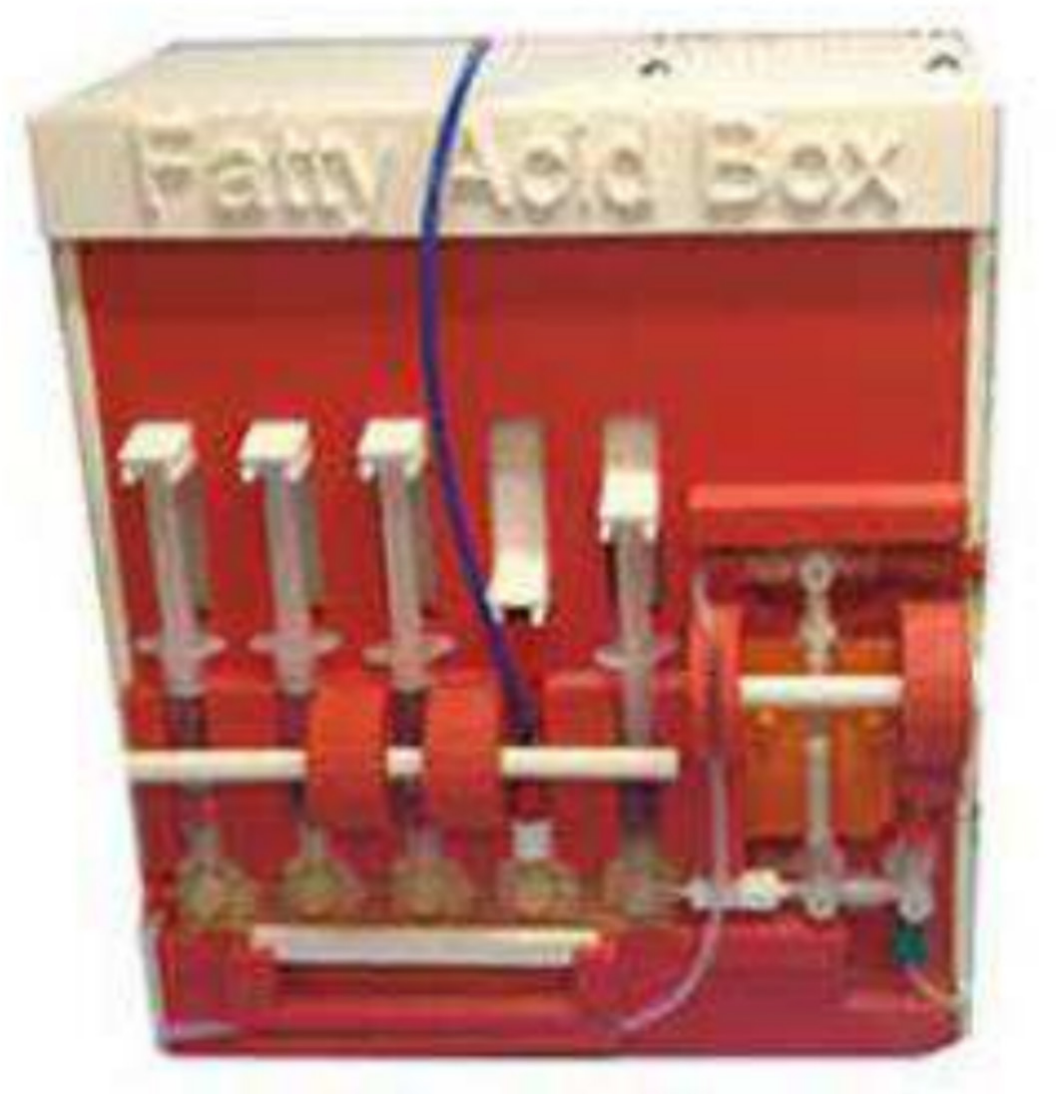
3D printed module.

Purpose‐built 3D‐printed modules offer a compact, cost‐effective, and highly customizable automated system for PET radiotracer synthesis (Figure 3). These modules can be optimized for each tracer and integrate only the necessary reactors, purification systems, and fluid‐handling mechanisms into a small physical footprint. The reported system is optimized for the production of carbon‐11 fatty acids and offers significant advantages in reproducibility, reduced radiation exposure, and adaptability to diverse chemistries.

### 3.3 Scintomics

Scintomics modules differ from the standard cassette module offerings in that they offer a versatile module design composed of submodule components [20]. Although these modules are not as widespread as some of the other modules, they can be arranged in advantageous ways to support both carbon‐11 cassette‐based radiosynthesis, as well as other isotopes.

### 3.4 GE Healthcare FASTlab

**FIGURE 4:**
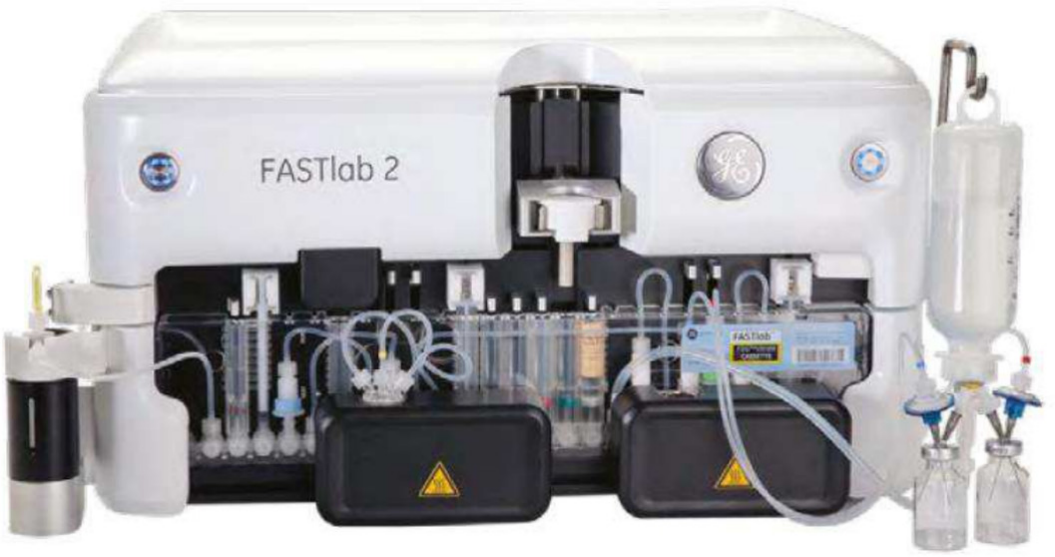
GE healthcare FASTlab 2.

The GE FASTlab platform is one of the most widely utilized radiosynthesis platforms available, with over 1000 installed worldwide. It utilizes a 25‐position cassette, which has three built‐in syringes (1 mL, 2 × 6 mL), and six vial positions (one of which can be used as a spike for a water bag or other bag) (Figure 4). The module can supply both vacuum and inert gas pushes, which are controlled along with the values by a computer. The FASTlab has been extensively utilized for fluorine‐18 radiosyntheses and gallium‐68 manufacturing, as well as more limited uses for other radiometals. There is one report of its use for carbon‐11 radiochemistry to prepare [^11^C]PIB.

### 3.5 iMiDEV Microfluidic Module

The iMiDEV microfluidic system is an automated radiochemistry platform for the synthesis of PET radiotracers, including those labeled with carbon‐11. Its compact design incorporates microfluidic reactors, solid‐phase extraction (SPE) units, and integrated fluid handling, enabling precise control of reaction conditions while minimizing reagent consumption and synthesis time [21].

### 3.6 Eckert & Ziegler Modular‐Lab PharmTracer [22]

The E&Z Modular‐Lab platform is a cassette‐based radiochemistry system designed for automated production of PET tracers, including several carbon‐11 radiopharmaceuticals. Single‐use cassettes are available for the generation of [^11^C]MeI, as well as for downstream synthesis of tracers such as [^11^C]choline, [^11^C]acetate, [^11^C]methionine, and [^11^C]raclopride. Some cassettes incorporate SPE purification, whereas others, like [^11^C]raclopride, include integrated HPLC purification. The system offers cGMP‐compliant workflows suitable for routine clinical use.

## 4 Developed Radiopharmaceutical on Cassette

### 4.1 [^11^C]Acetate

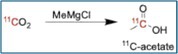

[^11^C]Acetate is widely used as a PET tracer for imaging oxidative metabolism, particularly in cardiac and oncologic applications. In tumors, it reflects lipid synthesis via the citric acid cycle, whereas in myocardium, it serves as a substrate for β‐oxidation. Its rapid clearance and low background make it a valuable tool in differentiating malignancy from benign processes, especially in prostate and hepatocellular cancers. [23, 24] Traditionally, it has been prepared through either manual [25] or fixed‐flow [7, 26] radiosyntheses. In 2017, Sai and coworkers published a cassette‐based automated radiosynthesis on the Trasis AIO [16]. The synthesis was carried out by [^11^C]carboxylation of MeMgCl in THF. After the trapping of the [^11^C]CO_2_ with the Grignard reagent at rt, the reaction was quenched with aqueous acetic acid solution and purified by passing the radioactive reaction mixture through activated PS‐H^+^, PS‐AG^+^, and PS‐OH^−^ ion‐exchange column resins. The final [^11^C]acetate was eluted from PS‐OH^−^ resin with aqueous citrate buffer solution into the final vial through a sterilizing filter to give a 51% activity yield and a 98% radiochemical purity.

In the 3D‐printed module [19], [^11^C]CO_2_ is trapped on a zeolite‐based SPE cartridge. Following trapping, [^11^C]CO_2_ is reacted with methylmagnesium bromide to yield [^11^C]acetate. The product is purified using SPE, with the final compound eluted into a sterile vial through a 0.22‐μm filter. This method achieves an activity yield of 62.8% ± 8.3% and a radiochemical purity of 97.5 ± 1.0%, with a total synthesis time of approximately 5.3 min.

### 4.2 [^11^C]Propionate

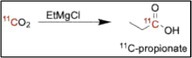

[^11^C]Propionate is a short‐chain fatty acid PET tracer used to investigate propionate metabolism, which plays a role in lipid biosynthesis and gluconeogenesis. Though less common than acetate, it has potential for probing intermediary metabolism in metabolic disorders and certain cancers. [^11^C]Propionate is synthesized using [^11^C]CO_2_ and ethylmagnesium bromide in a manner similar to [^11^C]acetate synthesis [19]. The reaction occurs on a zeolite cartridge and is followed by SPE purification. The product is eluted into a sterile vial with radiochemical purity exceeding 99.7% ± 0.2%. The decay‐corrected activity yields an average of 24.4% ± 5.1%, with a synthesis time of approximately 5.1 min.

### 4.3 [^11^C]Palmitate

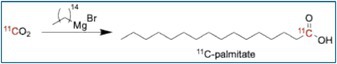

[^11^C]Palmitate is a long‐chain fatty acid analog used to measure myocardial fatty acid uptake and oxidation. It has also been used in metabolic studies of diabetes and obesity. Its longer chain length compared with acetate allows for evaluation of distinct metabolic pathways relevant to lipid homeostasis [27]. [^11^C]Palmitate, a long‐chain fatty acid tracer, is synthesized by reacting [^11^C]CO_2_ with pentadecylmagnesium bromide on a zeolite‐based cartridge [19]. The reaction proceeds at room temperature, followed by washing and SPE purification steps. The purified [^11^C]palmitate is eluted with a solution of acetic acid and ethanol. This process achieves a decay‐corrected activity yield of 57.2% ± 12.4% and a radiochemical purity of 96.7% ± 0.8%, with a synthesis time of approximately 6.5 min.

### 4.4 [^11^C]Acetoacetate

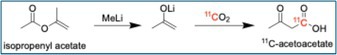

[^11^C]Acetoacetate (AcAc) is a ketone body tracer used to assess ketone metabolism in the brain, heart, and kidneys [28]. It has been applied in studies of aging, neurodegeneration, and cardiac metabolism, often alongside [^11^C]acetate to differentiate metabolic substrate preference in various disease states. [^11^C]AcAc can be used as a measure of ketone metabolism, especially in the brain [24, 29]. In 2017, Sai and colleagues reported the automated radiosynthesis of [^11^C]AcAc. Starting with a solution of methyl lithium in diethyl ether, isopropenylacetate was manually added under inert conditions, which was then mounted on the cassette/AIO [17]. The solution was cooled to −25°C (±15°C) via liquid nitrogen, controlled from the module. The cyclotron‐produced [^11^C]CO_2_ was bubbled through the solution, and the mixture was allowed to react for 6 min at which time it was hydrolyzed with water. The [^11^C]AcAc was purified via solid phase purification and eluted with a citrate buffer through a sterilizing filter. The radiosynthesis gave a 35% activity yield with the radiochemical purity of > 95% and a synthesis time of 16 min.

### 4.5 [^11^C]Methionine

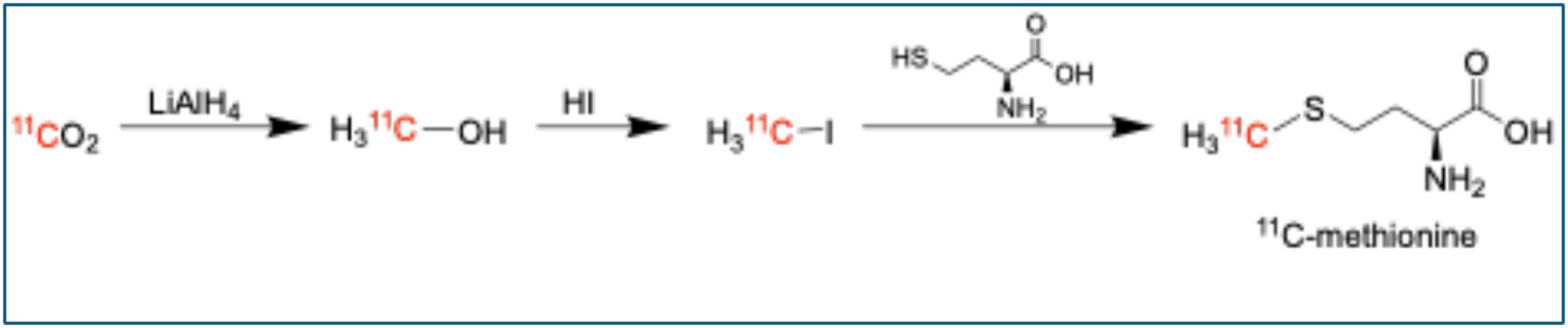

*L*‐[^11^C]Methionine is a PET radiotracer used to assess amino acid transport and protein synthesis, particularly in brain tumors. It offers improved tumor‐to‐background contrast compared with [^18^F]FDG, making it especially useful for imaging low‐grade gliomas and recurrent glioblastoma. On the AIO module, L‐homocysteine is preloaded onto a tC18 cartridge, and the cassette‐based system generates [^11^C]MeI via the wet method. The [^11^C]MeI is transferred to the cartridge to form [^11^C]methionine. The labeled product is eluted with phosphate buffer and saline through a sterilizing filter into the product vial, yielding > 20% activity yield in 15 min with 98% radiochemical purity and enantiomeric purity > 99.5%. The iMiDEV module converts [^11^C]CO_2_ into [^11^C]CH_3_I, which is delivered to a reactor preloaded with L‐homocysteine thiolactone and HLB resin [30]. The radiolabeling reaction is complete in approximately 3 min. After SPE purification, the final product is eluted with phosphate‐buffered saline (PBS) and sterile filtered. The synthesis achieves a radiochemical yield of 84% ± 4% (decay‐corrected), with radiochemical purity of > 96%, with a synthesis time of 18 min.

### 4.6 [^11^C]Choline

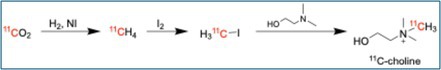

[^11^C]Choline is a PET tracer for imaging phospholipid metabolism and cell membrane synthesis, frequently used in prostate cancer and certain brain tumor studies. Its rapid uptake and fast clearance from nontarget tissues enhance diagnostic accuracy. Cassette‐based synthesis on the AIO module uses dimethylaminoethanol preloaded onto a CM cartridge. The [^11^C]MeI generated from [^11^C]CO_2_ is delivered to the cartridge where the labeling occurs. Following reaction, the cartridge is washed with ethanol and water, and the product is eluted with PBS, providing > 30% activity yield in 15 min with > 98% radiochemical purity. On the iMiDEV module, [^11^C]CO_2_ is converted to [^11^C]CH_3_I in a precursor module and transferred to a reactor preloaded with dimethylaminoethanol on CM resin [30]. The methylation reaction is conducted under ambient temperature conditions. SPE purification removes unreacted precursor and impurities, and the purified [^11^C]choline is eluted into a sterile saline solution. The process delivers a radiochemical yield of 66% ± 2%, with radiochemical purity of > 99%, in approximately 24 min.

### 4.7 [^11^C]PIB

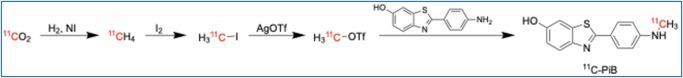

[^11^C]Pittsburgh Compound B (PIB) has been the gold standard for measurement of β‐amyloid burden for several decades [31]. Not only does it provide excellent images, but its short half‐life allows for a second scan on the same subject later in the day with a tau or other imaging agent of choice. It enables early diagnosis, disease staging, and longitudinal monitoring of therapeutic interventions in Alzheimer's disease. Until recently, [^11^C]PIB has been exclusively prepared for clinical research on fixed‐flow automated synthesis modules, with either a reactor or “loop”‐based approach [7]. In 2023, Sai and colleagues reported the automated synthesis of [^11^C]PIB on the Trasis AIO, utilizing the AIO for both preparation of the [^11^C]MeOTf and the radiosynthesis of [^11^C]PIB [12]. The [^11^C]MeOTf was generated as previously discussed and passed through a C18 sep‐pak that had been loaded with the precursor. After the maximal amount of [^11^C]MeOTf was transferred, the reaction was continued for 50 s, at which time the sep‐pak was eluted with 60% MeCN/H_2_O and injected onto the HPLC for purification. The HPLC fraction was collected, diluted with sterile water, and trapped on a second C18 sep‐pak. This sep‐pak was rinsed with sterile water, eluted with ethanol, and diluted with saline. The activity yield was 9.8% ± 1.7% and the molar activity was 57 ± 18 GBq/μmol for a 25‐min synthesis.

In 2017, Boudjemeline and coworkers published a cassette‐based synthesis of [^11^C]PIB on the Scintomics module series [32]. This work was groundbreaking for the radiosynthesis on cassette as it demonstrated the labeling of 6‐OH‐BTA‐0 with [^11^C]MeOTf on a sep‐pak that was preloaded with the precursor. They utilized a Synthra C‐11 module to convert cyclotron‐produced [^11^C]CO2 to [^11^]MeOTf, which was then passed over the precursor‐loaded sep‐pak. After radiolabel incorporation, they then used the same sep‐pak for solid‐phase purification by elution with increasing concentrations of ethanol in an acetate buffer. The product was then eluted from the sep‐pak and formulated to give the desired [^11^C]PIB in excellent activity yield (23.6% ± 2.7% from [^11^C]MeOTf), radiochemical purity (98.1% ± 0.8%), activity (190 ± 90 GBq/μmol), and meeting the other quality metrics required for cGMP use. The authors also note that the acetone used to dissolve the precursor can degrade the polycarbonate cassette manifold; however, this can be alleviated by the use of specialized manifolds.

**FIGURE 5:**
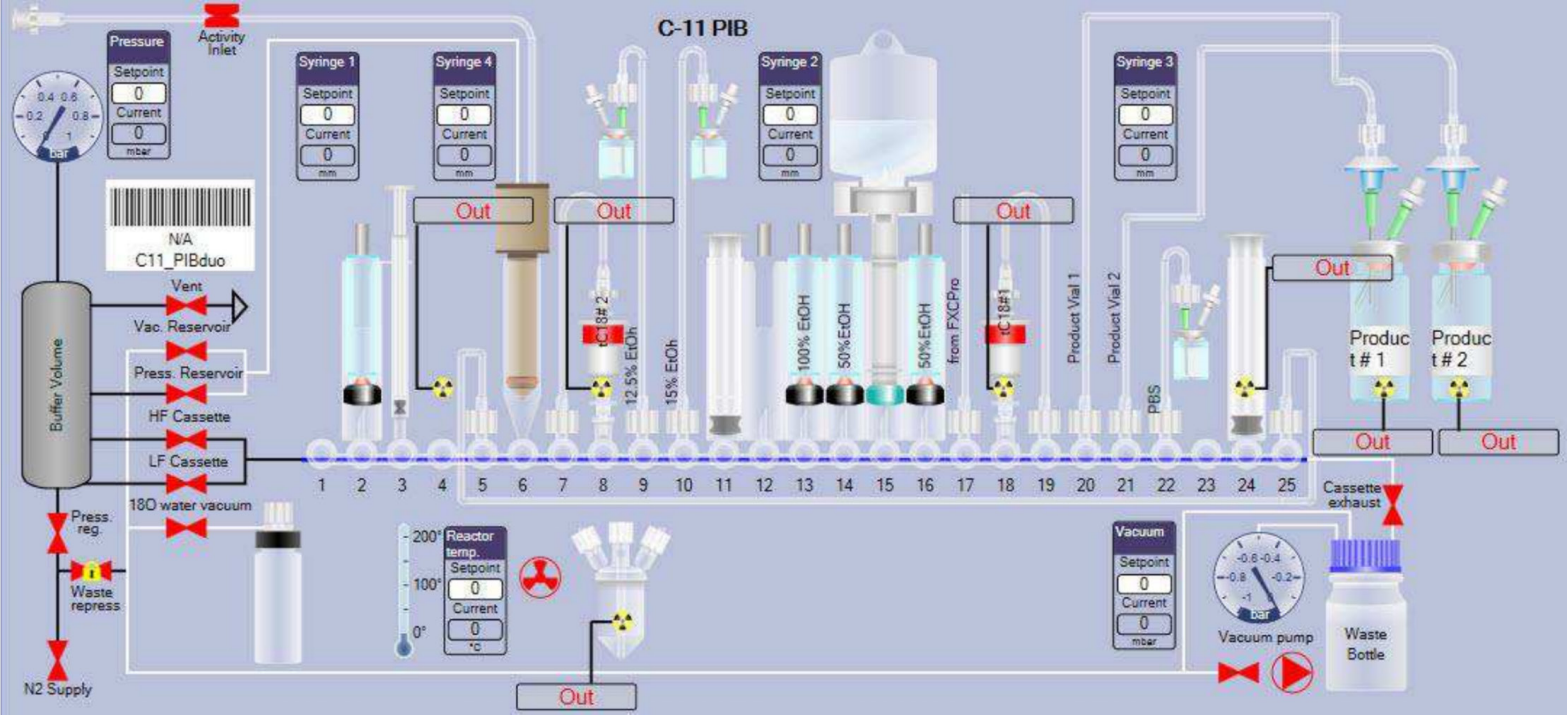
FASTlab dual [^11^C]PIB cassette layout.

The most recent publication of [^11^C]PIB on a cassette module was published by Nair and colleagues in 2024 (Figure 5) [33]. This radiosynthesis used [^11^C]MeOTf produced via the gas‐phase method on a TRACERlab FXc Pro and delivered to the FASTlab cassette. Starting with the concept developed by Boudjemeline [32], the process was optimized on the FASTlab to provide 2.58–3.85 GBq with excellent radiochemical purity (> 98%) and high molar activity (825 ± 124 GBq/μmol) in a 16‐min radiosynthesis (from [^11^C]MeOTf delivery start) without the use of HPLC purification. The process was validated for use in clinical research and accepted by the FDA. Additionally, Nair and colleagues developed a cassette that would allow for a second production of [^11^C]PIB up to 6 h after the end of the first production. This allows for multiple productions of [^11^C]PIB in a single day on a single module/hotcell without an operator having to expose themselves to radioactivity to clean a hot module.

### 4.8 [^11^C]LY2795050

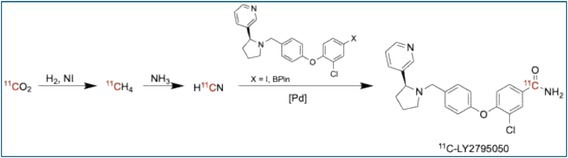

[^11^C]LY2795050 is a selective antagonist for the κ‐opioid receptor, enabling in vivo studies of the endogenous opioid system. It has been used to study mood disorders, pain, and addiction and supports kinetic modeling with well‐characterized binding dynamics in the human brain [34]. Sai and colleagues published the synthesis on the Trasis AIO using a palladium‐mediated cyanation using [^11^C]cyanide at 85°C for 3 min, followed by base hydrolysis, HPLC purification, and SPE reformulation to give [^11^C]LY2795050 in 28% radiochemical yield with moderate molar activity (96.2 GBq/μmol) and > 99% radiochemical purity over a 40 min radiosynthesis [18].

### 4.9 [^11^C]Flumazenil

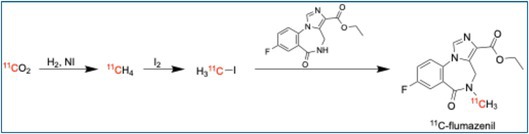

[^11^C]Flumazenil binds to the benzodiazepine site of the GABA_A receptor and is widely used to image inhibitory neurotransmission. It is particularly valuable in epilepsy, anxiety, and hepatic encephalopathy studies. Changes in [^11^C]Flumazenil binding can reflect neuronal loss or altered receptor density [35]. On the iMiDEV system, the synthesis begins with cyclotron‐produced [^11^C]CO_2_, converted into [^11^C]MeI in an adjacent module [36]. The [^11^C]CH_3_I is transferred to a reactor preloaded with the desmethyl flumazenil (DMFZ) precursor. Methylation proceeds at 50°C–100°C, to produce [^11^C]flumazenil. The crude product undergoes SPE purification, with final formulation in saline and ethanol. The synthesis achieves a radiochemical yield of > 25%, with high radiochemical purity (> 98%), good molar activity (1912 ± 552 GBq/μmol), and a synthesis time of approximately 25 min.

### 4.10 [^11^C]L‐Deprenyl

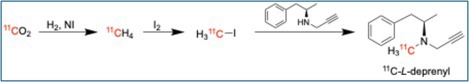

[^11^C]L‐Deprenyl targets monoamine oxidase B (MAO‐B), which is upregulated in reactive astrocytes. It serves as a marker of neuroinflammation and has applications in Parkinson's disease, Alzheimer's disease, and brain injury research. The synthesis on the iMiDEV system starts with [^11^C]CO_2_, converted to [^11^C]CH_3_I using the TracerMaker module [36]. The [^11^C]CH_3_I is transferred to a reactor containing the L‐Deprenyl precursor. Methylation occurs at room temperature, followed by SPE purification to isolate [^11^C]L‐Deprenyl. The final product is eluted into a sterile vial and filtered. The process achieves a radiochemical yield > 60% and a radiochemical purity > 95%, with a total synthesis time of approximately 20–25 min.

### 4.11 [^11^C]Butanol

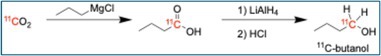

[^11^C]Butanol is an important radiotracer for studying blood perfusion, cerebral blood flow, blood‐brain perfusion, and blood‐brain barrier status [37]. Although this tracer was first prepared 50 years ago, its radiosynthesis has recently been reported on the GE FASTlab 2 module [38]. The [^11^C]CO_2_ was introduced via bubbling into a solution of *n*‐propylmagnesium chloride. Immediately after a lithium aluminum hydride solution was transferred to the reaction mixture to reduce the acid to butanol. The crude reaction was then dried down, quenched with HCl, diluted with water, and purified on a C18 sep‐pak. The [^11^C]butanol was produced in 4%–8% activity yield with a radiochemistry purity of > 95% in 21 min.

### 4.12 Radiosyntheses Summary

The radiosyntheses described above are successful manufacturing processes on cassette‐based modules (Table 1). These radiosyntheses utilize a number of different automated modules and all give activity yields acceptable for the production of clinically relevant doses. Molar activity is also a critical variable, and all of the reported values are acceptable for clinical research, although there is a large range; the fatty acids, methionine, and choline do not report values since these are transported radiopharmaceuticals and not targeting receptors where high molar activities are critical.

**TABLE 1:** Summary of radiosyntheses.

Tracer	Module	AY	RCP	Am (GBq/μmol)
Acetate	Trasis AIO	51%	98%	—
Acetate	3D‐Printed	63%	98%	—
Propionate	3D‐Printed	24%	> 99%	—
Palmitate	3D‐Printed	57%	97%	—
Acetoacetate	Trasis AIO	35%	95%	—
Methionine	iMiDEV	84%^a^	96%	—
Choline	iMiDEV	66%^a^	> 99%	—
PIB	Trasis AIO	10%	98%	57
PIB	Scintomics	24%	98%	190
PIB	FASTlab	10%	98%	825
LY2795050	Trasis AIO	28%^a^	99%	96
Flumazenil	iMiDEV	13%	98%	1912
Deprenyl	iMiDEV	4%	98%	1463
Butanol	FASTlab	4‐8%	> 95%	—

^a^ Radiochemical yield.

## 5 Future of Carbon‐11 Automated Radiosynthesis

For most of its history, carbon‐11 radiopharmaceuticals have been prepared by manual, semiautomated, or fixed‐flow automated modules. With the new developments in carbon‐11 on cassette modules, it is important to consider what the optimal manufacturing strategy is. We feel that for chemistry development, fixed‐flow modules offer a superior choice given their simpler programming and fewer potential developmental hurdles. However, over the course of a radiopharmaceutical life cycle, it becomes advantageous to move to a cassette‐based module, even if the carbon‐11 synthon needs to come from an external module. A single carbon‐11 synthon module (such as the TRACERlab FX2 MeI) can supply up to four destination modules that perform the labeling incorporation and purification steps. With the new methodology described in this review, cassette modules now have the capability to perform both carbon‐11 radiosyntheses as well as other radionuclides. For manufacturing centers, like academic centers, where there is a wide variety of radiopharmaceuticals produced with limited resources, such as hotcell space, this means a greater economy of scale for diverse radiopharmaceutical production capabilities. From a cGMP perspective, using a single‐use preassembled cassette provides a highly reproducible process that requires minimal setup time. The use of a disposable, typically commercially available, cassette with the cartridges, tubing, and reagents already assembled/prepared is a straightforward way to increase regulatory compliance. Fixed‐flow systems require a validated cleaning process to be performed in between radiosyntheses, increasing the regulatory compliance burden along with operator set‐up time. Another factor that needs to be considered is the cost‐benefit analysis of a fixed‐flow system versus a cassette system. At first glance, the costs for a fixed‐flow system appear to be cheaper, not requiring the purchase of the cassette skeleton or disposable tubing needed for a cassette module; however, these costs become more equitable when the cleaning materials and time are factored in, along with the much simpler setup with a cassette system. Lastly, the engineering possibilities on a cassette are simply greater than those of a fixed‐flow system. For example, on the [^11^C]PIB process [32, 33], the purification takes place on a separation cartridge using multiple elutions with specific mixtures, volumes, and flow rates; this is not possible with the current generations of fixed‐flow systems. With the highly beneficial use of a SPE approach over HPLC purification, this additional cassette capability can be very useful.

Going forward, it is likely that cassette‐based modules will be increasingly utilized for carbon‐11 radiosyntheses. Given that the most valuable resource in most manufacturing facilities is hotcell space and the number of modules that can be accommodated, being able to have modules that can switch between carbon‐11 and other radionuclides quickly is highly desirable. Furthermore, the simpler production day workflow reduces the burden on the operators and minimizes potential problems. Although fixed‐flow modules for carbon‐11 are critical for the development of new chemistries and new radiopharmaceuticals, as a radiopharmaceutical advances through its life cycle, a cassette‐based radiosynthesis offers many benefits.

## 6 Conclusion

The production of carbon‐11 radiopharmaceutical synthesis on cassette‐based modules represents a significant advancement in radiochemistry, streamlining the production process while enhancing reproducibility and operational efficiency. The transition from fixed‐flow synthesis to preassembled, single‐use mitigates many of the challenges associated with traditional methods, including variability in module setup, contamination risks, and labor‐intensive cleaning procedures. A broad range of carbon‐11 radiotracers has been made on cassette‐based modules, including some of the most commonly produced carbon‐11 tracers, such as [^11^C]PIB, [^11^C]methionine, [^11^C]choline, and [^11^C]acetate.

Despite these advancements, several limitations remain. The reliance on proprietary cassettes can increase costs and limit flexibility, particularly for novel tracer development. Additionally, although cassette‐based automation improves standardization, certain complex multistep syntheses may still necessitate customized setups or alternative strategies. Although many of the methods described above rely on external production of the carbon‐11 synthons, a cassette‐based method for this has been developed on the Trasis AIO. Further research into optimizing cassette designs, enhancing radiochemical yields, and expanding the chemical versatility of automated platforms will be critical for future developments.

## Conflict of Interest

GE Healthcare provides funding and resources for ongoing research collaborations with Dr. Rosenberg including project funding, salary support, and sponsored travel.

## Data Availability

Data sharing is not applicable in this article as no datasets were generated or analyzed during the current study.
